# Isolation of dissolved organic matter from aqueous solution by precipitation with FeCl_3_: mechanisms and significance in environmental perspectives

**DOI:** 10.1038/s41598-023-31831-1

**Published:** 2023-03-20

**Authors:** Jie Zhang, Khan M. G. Mostofa, Xuemei Yang, Mohammad Mohinuzzaman, Cong-Qiang Liu, Nicola Senesi, Giorgio S. Senesi, Donald L. Sparks, H. Henry Teng, Longlong Li, Jie Yuan, Si-Liang Li

**Affiliations:** 1 Institute of Surface-Earth System Science, School of Earth System Science, Tianjin University, 92 Weijin Road, Tianjin, 300072 China; 2 Tianjin Key Laboratory of Earth Critical Zone Science and Sustainable Development in Bohai Rim, Tianjin University, 92 Weijin Road, Tianjin, 300072 China; 3 Institute of Ecology, College of Urban and Environmental Sciences, Peking University, Beijing, China; 4 Department of Environmental Science and Disaster Management, Noakhali Science and Technology University, Noakhali, Bangladesh; 5 Dip.to di Scienze del Suolo, della Pianta e degli Alimenti, Università Degli Studi Di Bari “Aldo Moro”, Via G. Amendola 165/A, 70126 Bari, Italy; 6 CNR - Istituto per la Scienza e Tecnologia dei Plasmi (ISTP) - Sede Di Bari Via Amendola, 122/D, 70126 Bari, Italy; 7 Department of Plant and Soil Sciences, Delaware Environmental Institute, University of Delaware, Newark, DE 19716-7310 USA; 8 College of Resources and Environment, Xingtai University, Quanbei East Road 88, Qiaodong District, Xingtai City, Hebei Province China; 9 Haihe Laboratory of Sustainable Chemical Transformations, Tianjin, 300192 China

**Keywords:** Biogeochemistry, Environmental sciences

## Abstract

Ferric ions can bind strongly with dissolved organic matter (DOM), including humic acids (HA), fulvic acids (FA), and protein-like substances, whereas isolation of Fe-DOM precipitates (Fe-DOM_P_) and their biochemical characteristics remain unclear. In this work FeCl_3_ was used to isolate DOM components from various sources, including river, lake, soil, cow dung, and standard tryptophan and tyrosine, through precipitation at pH 7.5–8.5. The Fe-DOM_P_ contribute to total DOM by approximately 38.6–93.8% of FA, 76.2% of HA and 25.0–30.4% of tryptophan and tyrosine, whilst fluorescence spectra allowed to monitor/discriminate the various DOM fractions in the samples. The relative intensity of the main infrared peaks such as 3406‒3383 cm^−1^ (aromatic OH), 1689‒1635 cm^−1^ (‒COOH), 1523–1504 cm^−1^ (amide) and 1176–1033 cm^−1^ (‒S=O) show either to decline or disappear in Fe‒DOM_P_. These results suggest the occurrence of Fe bonds with various functional groups of DOM, indicating the formation of π–d electron bonding systems of different strengths in Fe‒DOM_P_. The novel method used for isolation of Fe-DOM_P_ shows promising in opening a new frontier both at laboratory and industrial purposes. Furthermore, results obtained may provide a better understanding of metal–organic complexes involved in the regulation of the long-term stabilization/sequestration of DOM in soils and waters.

## Introduction

Soil organic matter (SOM) is composed primarily of humic substances (HS), i.e. humic acids (HA), fulvic acids (FA) and protein-like substances, a portion of which is leaching and contributing to river/lake/sea as allochthonous HS^1,2^, whereas autochthonous HS occurring in freshwaters are derived from photosynthetically-originated planktonic communities, i.e. the phytoplankton^3–7^. Furthermore, HA-like or FA-like substances in various organic wastes such as cow dung samples are freshly produced from microorganisms present in these materials, whereas the initial presence of tyrosine-like substances mostly disappear^8,9^.

Ferric ions are well known to form stable complexes with (*i*) soil HS, including HA, FA and protein-like substances, which primarily affect the stability and preservation of soil and sediment organic matter (SOM)^10–14^; and (*ii*) terrestrial and autochthonous dissolved organic matter (DOM), including FA, HA, tryptophan and tyrosine that are commonly detected in surface waters^6,15–18^. These versatile properties of ferric ions (Fe^3+^) control many important biogeochemical processes and functions, including: (*i*) formation of organo-mineral compounds in soils and sediments^10–14^ (*ii*) flocculation and coprecipitation of HS and, in turn, their deposition in estuarine and coastal seawaters after input from riverine transportation^19–22^; (*iii*) siderophore complexation by prokaryotes, which influences primary productivity in natural waters via photosynthesis and nitrogen fixation^23–27^; and (*iv*) the photo-Fenton reactions, which regulate the photochemistry and redox reactions in surface waters^28–31^. Moreover, about 99% of Fe in marine waters is shown to be strongly complexed with DOM^32,33^. Ideally, DOM precipitation with FeCl_3_ can provide four important novel/unique information on biogeochemical processes occurring in soils and waters. Firstly, soil HS components are well-known to form complexes with Fe^3+^ ions either via co-precipitation and adsorption/co-sorption^34–38^ or chemical bonding between HS functional groups and soil minerals^39,40^. In particular, Reiller et al. showed that HA of high molecular weight can preferentially adsorb to hematite^34^, whilst Newcomb et al. measured directly the binding between organic ligands with soil minerals^40^. However, the precipitation mechanisms of soil HS by Fe^3+^ by forming a π‒d electron bonding system between the HS functional groups and d-orbitals of Fe remain unclear. Secondly, Fe^3+^ shows to sink HS in aquatic environments which typically occurs by trapping terrestrially derived DOM by Fe^3+^^20^, flocculation and coprecipitation^19–22,41^. Similarly, Fe^3+^ can form complexes with protein of extracellular polymeric substances (EPS) and siderophores of prokaryotes^23–26,42^, and may act as a key factor for their subsequent sinking in aquatic environments. However, the mechanisms by which functional groups of various DOM components, including amino acids (tryptophan and tyrosine), can effectively form complexes with d-orbitals of Fe^3+^ in terms of bonding formation mechanism is still unclear to date. Thirdly, FA is commonly isolated from aqueous solutions by the well-accepted method that uses organic-based XAD-8 resins^43–45^. However, a problem with XAD-8 resins is the possible incorporation of organic matter from the adsorbent which demands a careful resin cleanup^43,46^, the loss of a substantial portion of FA as shown by the 23–58% of dissolved organic carbon (DOC) recovered by precipitation and adsorption onto XAD-8 resin in clear water and 50–90% in colored surface waters^43,47^, and recovering of only the hydrophobic fraction of FA^43,48^. Furthermore, PPL (styrene divinyl benzene polymer) is also reported to be able to extract an average 43% of deep sea DOC and up to 65% of freshwater DOC^49^. Therefore, a novel extraction technique based on the use of Fe^3+^ ions appears very relevant to isolate diverse forms of FA from aqueous solutions as Fe-FA precipitates (Fe-FA_P_). In particular, FA isolated in a pure form as Fe-FA_P_ may provide different individual fractions of FA free of impurities, which cannot be isolated using XAD-8 resins. In this regards, Fe-FA_P_ are obtained based on the principle that supersaturation governs the appearance of precipitates^50,51^ and alkaline conditions may lead to form large aggregates that can settle under the influence of gravity^52^.

Therefore, a novel method based on the use of FeCl_3_ is developed in this work aiming to: (*i*) isolate FA from diverse environmental aqueous media, including rivers, lakes, soils and cow dungs, as Fe‒FA_P_; (*ii*) characterize them by FTIR and fluorescence excitation-emission (EEM) spectroscopy coupled with parallel factor (PARAFAC) modelling to assess their molecular nature and adsorption/complexation behavior, and propose tentative mechanisms for their formation; (*iii*) isolate HA, FA and protein-like substances from HA in alkaline extracts of soil and characterize the corresponding Fe^3+^ precipitates and remaining supernatants obtained by a series of successive precipitation steps by fluorescence EEM spectroscopy, and ascertain the precipitation of HA by FeCl_3_ (Fe-HA_P_); (*iv*) characterize the pH-8.5 re-dissolved Fe-HA_P_ isolated from the extracted HA sample in order to ascertain the occurrence of coprecipitation by HA; (*v*) isolate and characterize low molecular weight DOM, i.e., tryptophan and tyrosine, from their aqueous solutions to determine their Fe-DOM_P_; and (*vi*) propose the optimal conditions for the application of FeCl_3_ to precipitate DOM fractions from various aqueous solutions and discuss their relevance at environmental and industrial scales.

## Materials and methods

### Samples

The DOM fractions were isolated from the Baigu river and Jingye lake waters, a forest soil, and original and irradiated cow dung samples. The 38.5-km-long Baigou River features clear and unpolluted waters originating from the foothills of the Taihang Mountains in the northwest of Laiyuan County, Hebei Province, China. The sampling site was located in the upper part of the river, approximately 150-m from the middle section of the North Juma River. The Jingye lake is situated in the Weijin Road campus of Tianjin University, Tianjin, China, and its DOM was comprehensively characterized in earlier studies that showed its origin was mostly from photosynthetically-derived primary productivity^6^. The forest soil was collected under the Panshan deciduous forest, Tianjin city of north China and details on its sampling site, vegetation covers and major physico-chemical characteristics were reported previously^2^.

The cow dung samples were collected from a farmhouse located in a village in the Jixian district of Tianjin city. Besides the original sample, a cow dung sample irradiated for 1 day under natural sunlight was used in order to ascertain how does the DOM in cow dung transform under sunlight conditions. Raw cow dung samples without any treatment were shown to be primarily composed of high amounts of tyrosine^9^, with an increasing humified fraction during its full-scale vermicomposting^8^. The reason to study also an irradiated sample is based on the fact that during cattle grazing on the grass-field in day-time, cow dung is subject to irradiation by natural sunlight and mixing up with soil and rainwater. Thus, the study of one-day sunlight-irradiated cow dung is expected to provide useful information on the possible alteration of organic matter, and especially its FA fraction, in these conditions. Thus, the cow dung samples were dried in an oven at 60 °C, ground into fine particles, and then stored at − 20 °C until further processing.

Furthermore, two standard compounds, i.e. tryptophan and tyrosine purchased from BBI Life Sciences (Shanghai) and Shanghai Macklin Biochemical Co., Ltd, respectively, which are commonly detected in surface waters, rainwaters, glaciers, clouds and aerosols^5,6,15,53–57^, were used.

### Extraction of the liquid phase from forest soil and cow dung samples

The liquid phase was extracted from the forest soil using a number of subsequent steps (Fig. S1). In the first step, the ground and 0.2-mm-sieved soil sample was added with ultrapure water (18.2 MΩ·cm, Mill-Q, Millipore) at a soil/water ratio of 1:10, vortexed for 1 min in closed 500-mL brown bottles and then shaken for 6 h at 25 °C. The mixture was centrifuged for 20 min at 4000 rpm using a Thermo Fisher Scientific SORVALL ST 16 centrifuge to remove suspended solids. The supernatant solution was then filtered through a 0.45-µm membrane filter (GF/F type, Shanghai Xin Ya Purification Equipment Co. Ltd, China), whereas the remaining solid residue was extracted again with fresh ultrapure water for 1 h and the procedure described above applied again to obtain the supernatant solution that was mixed with the previous one and stored in a freezer at − 20 °C until further processing. This solution represents the soil water extract (W_e_).

The soil residue from water extraction was then subjected to alkaline extraction under N_2_ with a 0.1 M NaOH solution at a soil residue/alkaline solution ratio of 1:10 by shaking for 3 h at 25 °C. The mixture was then centrifuged as described above and the supernatant solution filtered through a 0.45-µm membrane filter (polytetrafluoroethylene membrane, PTFE, Shanghai Xin Ya Purification Equipment Co. Ltd, China). The remaining solid residue was extracted again with a fresh alkaline solution for 3 h and the procedure described above applied again to obtain the supernatant solution that was mixed with the previous one and stored in a freezer at − 20 °C until further processing. This solution represents the soil alkaline extract (A_e_).

The HA fraction of the forest soil was obtained by acidifying an aliquot of A_e_ at pH 2 under N_2_ at 4 °C. Many studies have shown that alkaline conditions (A_e_) may alter the molecular compositions of soil HA in the presence of O_2_ during its extraction from soil^45,58^, thus IHSS recommends to conduct the alkaline extraction in the presence of N_2_ gas to reduce any alterations^45^. After 24 h, the precipitated HA was centrifuged and freeze-dried. Cow dung samples were processed using the same procedure applied to the soil and described above. In this case, the precipitated HA fraction was redissolved in NaOH, adjusted to pH 8.5 and stored in a freezer at − 20 °C until further processing.

### Precipitation of DOM fractions by FeCl_3_

The Fe‒FA precipitates (Fe‒FA_P_) were obtained from the corresponding aqueous solutions by precipitation with FeCl_3_ according to the procedure outlined in the flow diagram in Fig. S2. Solid ferric chloride hexahydrate, analytical grade, 99.0% purity (Chemart Chemical Technology Co., LTD., AR, Tianjin, China) has been used in this study First, the surface water samples from Baigou River and Jingye Lake were filtered through a 0.45-μm GF-F type filter and the filtrates were acidified at pH 2 with HCl, kept for 24 h in a refrigerator at 4 °C, and then centrifuged for 20 min at 4000 rpm to remove HA. The supernatants were adjusted to pH 7.5–8.5 in 500 mL brown glass bottles, and then analyzed by fluorescence EEM spectroscopy to measure the initial fluorescence intensity of FA. Then, the FeCl_3_ solution (30 g L^−1^) was slowly added drop by drop to the supernatant solutions with simultaneous addition of NaOH to keep the pH at 7.5 ~ 8.5 and stirring gently using a magnetic stirrer. The pH values of 7.5–8.5 were chosen for three specific reasons: (*i*) Fe-DOM_P_ can form large aggregates under alkaline conditions^52^; (*ii*) the pH of the solution is decreasing constantly due to addition of Fe^3+^, thus it is needed to adjust the pH by NaOH continuously to maintain the chosen pH range; and (*iii*), this pH range is optimal for fluorescence measurements. The FeCl_3_ addition was stopped upon the appearance of Fe-FA_P_, but stirring was maintained for a further 5–10 min until the pH reached a constant value. The mixtures were then centrifuged for 20 min at 4000 rpm to separate the solid Fe-FA_P_, whereas the remaining supernatants were then adjusted to pH 7.5–8.5 and their fluorescence EEM spectra measured to monitor the approximate extent of FA precipitation in the first phase by comparing the actual intensity of FA peak M to the corresponding initial ones (at 285–315/373–416 nm). Peak M of FA is denoted as marine humic-like peak, and typically appears at shorter excitation-emission wavelengths (~ 280–320/370–420 nm) in comparison with peak C (~ 320–410/420–520 nm), whilst peak A occurs at shorter excitation and relatively longer emission wavelengths, and largely differ for HA (~ 270–280/440–520 nm), FA (~ 230–270/370–440 nm) and protein like substances (~ 220–230/385–420 nm) of both allochthonous and autochthonous origin^1,2,7,59,60^. About 3.0 mL of FeCl_3_ were needed for approximately 900 mL of supernatant to start the precipitation of Fe-FA_P_. The procedure was then repeated several times to precipitate subsequently Fe-FA_P_ fractions until the intensity of peak M of FA in the remaining supernatants reached a minimum. The solid Fe-FA_P_ fractions were then kept under inert N_2_ in a freezer at − 20 °C until further processing.

The Fe-HA precipitates (Fe-HA_P_) were obtained using the same isolation procedure used for Fe-FA_P_ (Fig. S2). Briefly, a 60-mg aliquot of soil forest HA was re-dissolved in 600 mL of 0.1 M NaOH and then adjusted to pH 8.5. The FeCl_3_ solution (30 g L^−1^) was slowly added drop by drop to a 400-mL aliquot of the HA solution, with simultaneous addition of NaOH to maintain the pH at 7.5 ~ 8.5, until the appearance of Fe-HA_P_. Approximately 1-mL of FeCl_3_ solution was needed to complete the precipitation. The Fe-HA_P_ fractions were isolated by centrifugation and the EEM fluorescence spectra of the corresponding remaining supernatants were measured to estimate the amounts of Fe-HA_P_ fractions. Then, the same procedure described above (Fig. S2) was applied to obtain the Fe-FA_P_ fractions from the remaining supernatants of HA. The dissolved organic carbon (DOC) content in the final remaining supernatants was measured to evaluate the amount of Fe-HA_P_ and Fe-FA_P_ fractions.

Similarly, a 40 mg aliquot of each standard tryptophan and tyrosine was dissolved in 400 mL aqueous solution at pH 8.5. The Fe-Tryptophan and Fe-Tyrosine precipitates (Fe-Tryptophan_P_ and Fe-Tyrosine_P_, respectively) were obtained by adding the FeCl_3_ solution to a 380-mL aliquot of each standard and using the same procedure described above for Fe-FA_P_ and Fe-HA_P_ (Fig. S2). Approximately 1 mL of FeCl_3_ solution was necessary to complete the precipitation of both compounds. In both cases, five subsequent precipitation steps were performed in order to obtain a higher amount of precipitate. The EEM fluorescence spectra of the remaining supernatants obtained after each step were measured to estimate the amount of Fe-Tryptophan_P_ and Fe-Tyrosine_P_ fractions on the basis of the corresponding fluorescence intensities. The DOC content in the final remaining supernatants was measured to evaluate the amount of Fe-Tryptophan_P_ and Fe-Tyrosine_P_. Noteworthy, the identical amounts of FeCl_3_ solution required in the first and subsequent steps of its drop by drop addition to samples to isolate Fe-DOM_P_ indicates that there is no excess amount of free Fe^3+^ in the remaining supernatant solutions. However, the Fe^3+^ concentration of the samples has not been measured in this study, which will focus for further study.

### Analytical protocols

The elemental C, H, N and S composition was measured using an elemental analyzer (Elemental Vario E.L. III, Germany) by placing an aliquot of each dried, ground and homogenized sample into a clean, carbon-free, pre-combusted tin boat placed on an autosampler rack assembly loaded onto the elemental analyzer. Sulfanilamide was used as a standard after every ten measurements. The O % was calculated by difference as O% = 100 − (C + H + N + S)%. The P% has not been measured in this study due to its low content^17,47^. The dissolved organic carbon (DOC) content was measured in triplicate for each sample using a combustion total organic carbon (TOC) auto-sampler analyzer (OI Analytical Aurora, Model 1030 W + 1088, USA). In this study, the blank value was below 0.15 mg L^−1^ (precision 15%), which was < 8.7% of the DOC concentration measured in all samples studied except two low DOC samples (11.6–15.2%).

Fourier transform infrared (FTIR) spectra were recorded using an IRAffinity-1S spectrometer (Shimadzu, Japan) that included a high-energy ceramic light source, a temperature-controlled, high-sensitivity (deuterated l-alanine triglycine sulfate: DLATGS) detector and a high-throughput optical element, with optimization of the electrical and optical systems. The IRAffinity-1S instrument achieves the highest signal/noise (SN) ratio in its class. A mixture of 2 mg of each dehydrated and freeze-dried sample was mixed homogeneously with 200 mg of dried KBr and pelletized by pressing under reduced pressure. Then the FTIR spectra of the pellets were recorded over the range 4000–400 cm^−1^ by averaging 30 scans at a 4 cm^−1^ resolution.

Fluorescence EEM spectra were measured by a fluorescence spectrophotometer (F-7000, Hitachi, Japan) using a procedure described previously^2,6^. Ultrapure (18.2 MΩ.cm) MQ water was used as the blank and measured every ten samples to check the performance of the instrument and ensure data quality. A 4-μg L^−1^ quinine sulfate (QS) solution in 0.01 mol L^−1^ H_2_SO_4_ was used to achieve fluorescence normalization and the fluorescence intensity of each sample was calibrated using the intensity of the QS (1 μg L^−1^ = 1 QS unit, QSU) peak at Ex/Em = 350/450 nm^2^. To avoid inner-filter effects and fluorescence quenching, each sample solution was diluted on the basis of the DOC concentration measured initially before EEM measurements^61^. Furthermore, the fluorescence intensity of each peak was rechecked and corrected using the common absorbance-based approach^62^. The Rayleigh and Raman peaks and the ultrapure water blank spectrum were subtracted from each experimental EEM spectrum using a home-made Excel program^2,6,63^. Preprocessed EEM data were then processed by the parallel factor (PARAFAC) model using the N-way toolbox for MATLAB^64^ as described elsewhere^3^. To avoid mixing of fluorescent components of different samples that could produce artifacts^63,65^, PARAFAC analysis was performed individually on each sample and on their remaining supernatant solution. Finally, non-negative constraints were applied to the PARAFAC model. The detailed procedure used for the PARAFAC analysis of EEM spectra was described previously^6,63,65^.

### Ethical approval

This manuscript does not involve to the researches regarding human participants, and also animals.

## Results and discussion

### Fe-FA_P_ from river, lake, soil and cow dung samples

**Figure 1:**
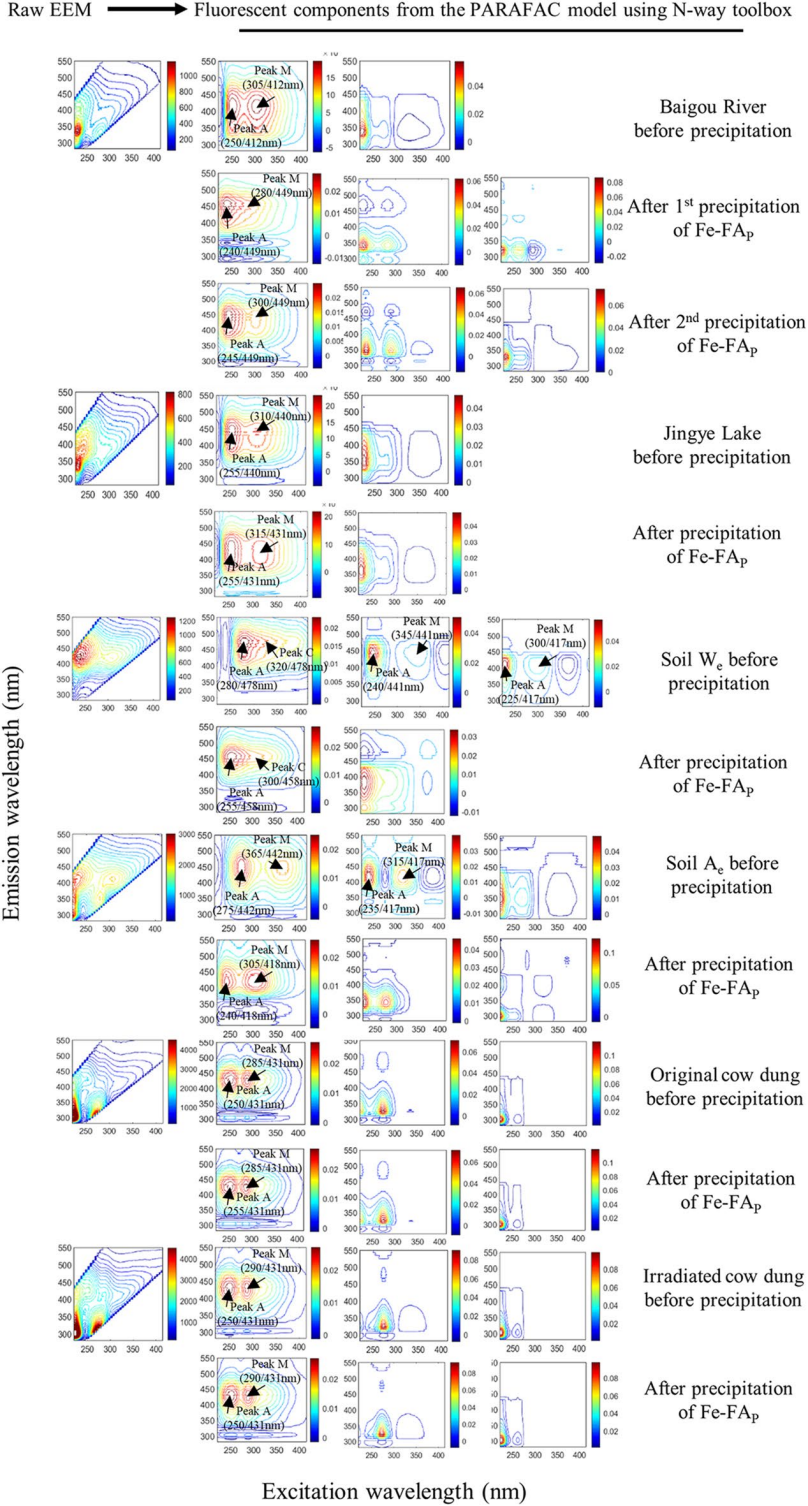
Original EEM spectra (left) and their fluorescent components identified by the EEM-PARAFAC model (right) of all individual aqueous samples from Baigou River, Jingye Lake, soil W_e_, soil A_e_, original cow dung and irradiated cow dung, and their remaining solutions (RS) after each repeated precipitation of Fe-FA_P_.. Two precipitations were performed for the Baigou River sample and one for all other samples.

The Fe‒FA_P_ obtained from the various environmental samples, i.e., Baigu river, Jingye lake, water-extracted (W_e_) and alkaline-extracted (A_e_) of the forest soil, and water extracts of original and irradiated cow dung, amounted respectively to 68.1%, 38.6%, 72.9%, 82.1%, 92.2% and 93.8% of FA (Table S1). The FA was monitored after each extraction step by measuring the fluorescence intensity of peak M of the remaining supernatants (Table S2). Peak M maxima varied in the ranges: 295–310/394–416 nm and 300–310/392–412 nm, respectively for soil W_e_ and A_e_; 315/403–410 nm and 315/400–411 nm, respectively for the first and the second precipitation from river water; 285–305/369–396 nm for lake water; and 315/402–415 nm and 312–315/402–415 nm, respectively for raw and irradiated cow dung (Table S2). Original EEM spectra (left-side) and their fluorescent components (right-side) identified by the EEM-PARAFAC model in all individual aqueous samples and their RS after each repeated precipitation of Fe-FA_P_ are shown in Fig. 1 and described in Table S3, and are very similar to those described in our earlier studies^2,6,7,9,57,63,65^. Furthermore, the loading figures corresponding to the fluorescent components identified by the EEM-PARAFAC model are presented in Figs. S3 and S4.

The FA precipitation as Fe-FA_P_ can be associated with two important biogeochemical phenomena: (*i*) the major fractions of terrestrial and autochthonous FA in riverine systems can form Fe-FA_P_ in coastal seawaters or estuarine environments commonly characterized by alkaline conditions^66^, which may sink a major fraction of FA in seawater environments as co-precipitates and/or flocculates^19,20,67,68^; and (*ii*) FA in soil can form Fe-FA_P_ in a complex state, Thus, FA in different environments such as streams, rivers, ponds, lakes, reservoirs, oceans, estuaries, ground waters, pore waters, rainwaters, clouds, snow, glaciers, soils, sediments and atmospheric aerosols can be easily isolated by precipitation with Fe^3+^ ions, which would allow their molecular-level analysis that can provide relevant information on their biogeochemical functions and processes in the corresponding environment/ecosystem. Noteworthy, Fe-FA_P_ isolated from various environmental samples represent the fraction of bulk DOM that associates with Fe^3+^ in the corresponding samples and thus cannot be directly compared with FA in pure forms.

### Soil Fe-HA_P_

**Figure 2:**
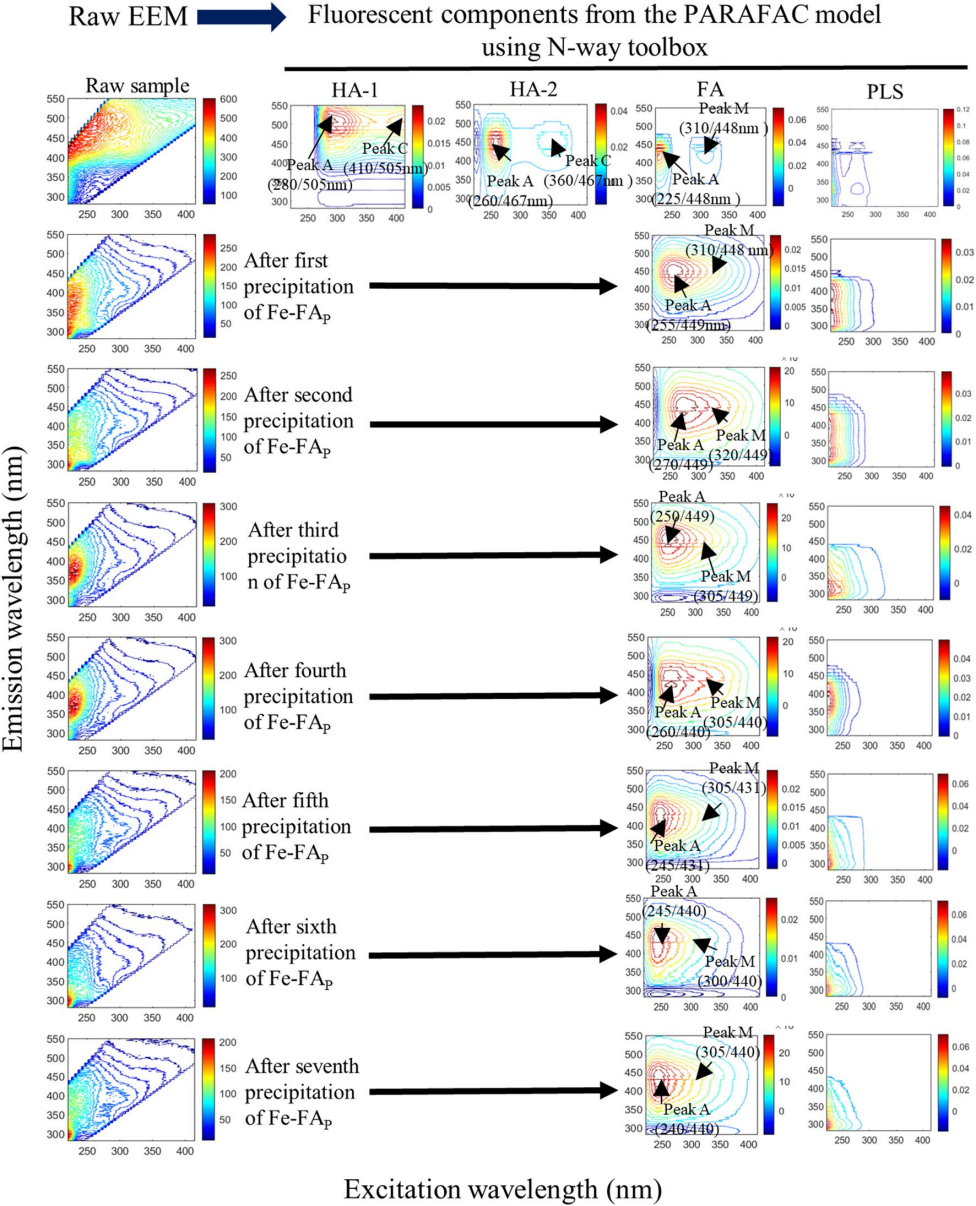
Original EEM spectra (left) and fluorescent components identified by the EEM-PARAFAC model (right) in humic acids (HA) and their remaining solutions (RS) after each repeated precipitation of Fe-HA_P_. The first line shows the original EEM spectra of the raw sample and the four fluorescent components identified by EEM-PARAFAC, which are, respectively, two humic acids (HA-1 and HA-2), fulvic acids (FA) and protein-like substances (PLS). Each subsequent row shows the original EEM spectra of the remaining solution (RS) after each of seven precipitation of Fe-FA_P_ and the fluorescent components identified by EEM-PARAFAC.

**Figure 3:**
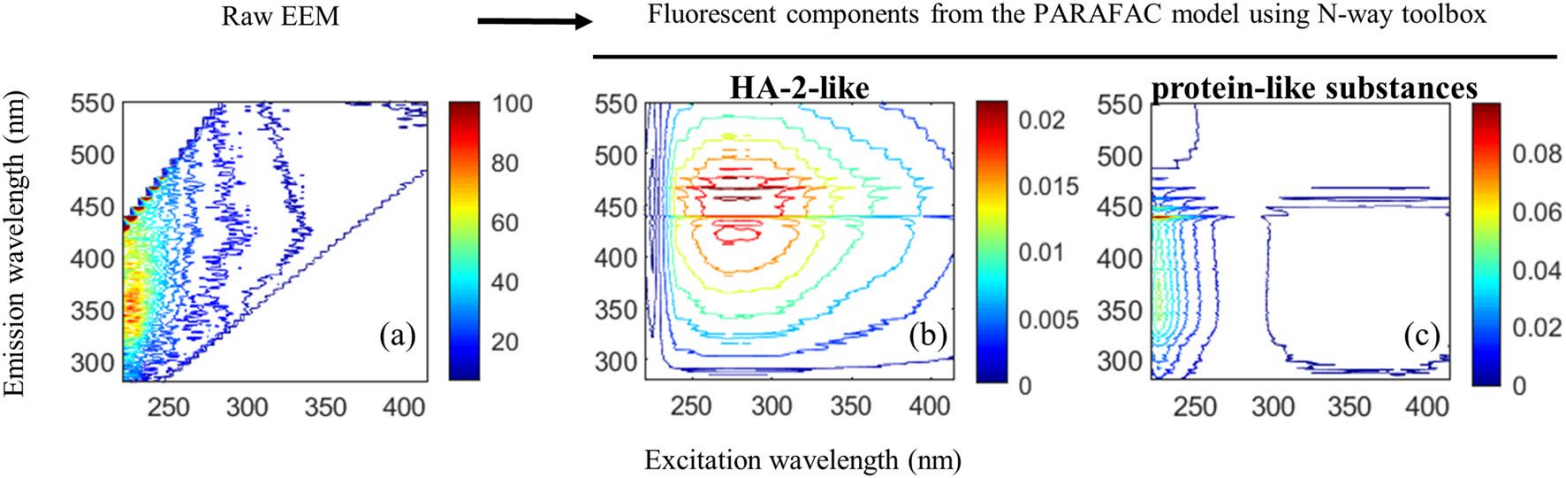
Original EEM spectra (left, **a**) and the two fluorescent components (**b**,**c**, right) identified by the EEM-PARAFAC model in isolated Fe-HA precipitates (Fe-HA_P_) after dissolution at pH 12.0.

**Figure 4:**
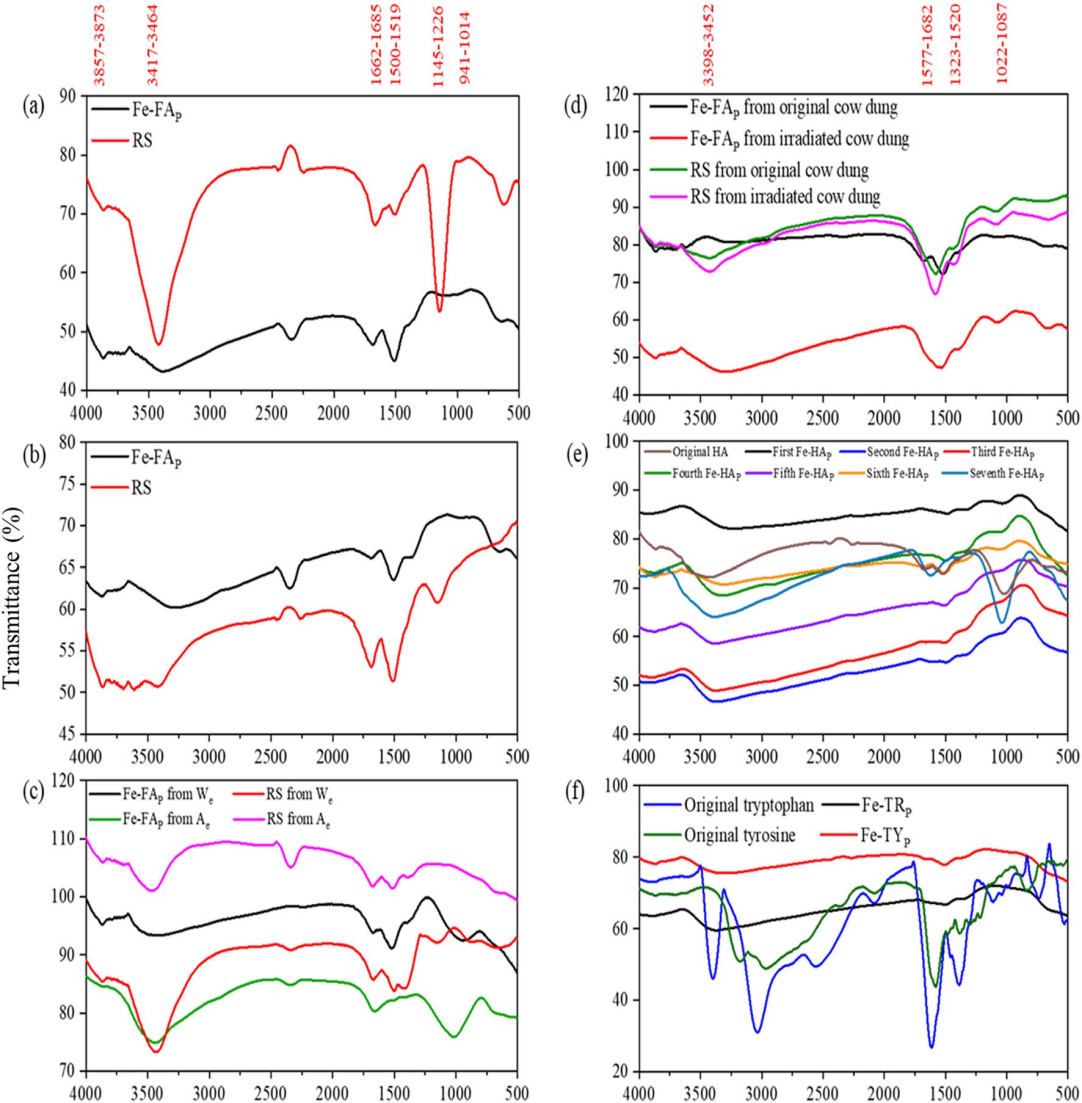
FTIR spectra of Fe‒fulvic acid precipitates (Fe‒FA_P_) and remaining solution (RS) after the final precipitation step of Fe‒FA_P_ from Baigou River (**a**), Jingye Lake (**b**), soil We and Ae (**c**), original and irradiated cow dung samples (**d**), original soil humic acids (HA) and Fe‒HA precipitates (Fe‒HA_P_) (**e**), and Tryptophan (TR) and Tyrosine (TY) and their FeCl_3_ precipitates (Fe‒TR_P_ and Fe‒TY_P_) (**f**).

The PARAFAC EEM images of soil HA and their corresponding peak maxima featured four fluorescent components, including HA-1, HA-2, FA and protein-like substances (Fig. 2, Table S3), which suggested the occurred co-precipitation of HA together with FA and protein-like substances at pH 2 from the forest soil A_e_. Similar components have been reported elsewhere^1,2^. Apparently, HA-1 and HA-2 in the soil A_e_ sample were precipitated and separated in the first step (Fig. 2), which suggested that two distinct types of HA would exist in the soil matrix. In the first extraction, 76.2% of total DOM was isolated as HA, whereas 41.0% of total DOM was isolated as FA by the following six extractions (Table S1). These results suggested that the main responsible for the formation of Fe-HA_P_ would be complexation by Fe^3+^ followed by co-precipitation. To confirm the occurrence of the co-precipitation of HA with protein-like substances, an aliquot of the Fe-HA_P_ sample was redissolved at pH 12.0 and analyzed by EEM fluorescence spectroscopy. Two fluorescent components from the HA-2-like and protein-like substances appear (Fig. 3), which suggest that co-precipitation of these two fractions occurred. Simultaneously, HA-1 would form strong complexes in Fe-HA_P_ that were not dissolved at pH 12.0, the evidence of which is provided from FTIR spectra that show the disappearance in Fe-HA_P_ of all IR peaks present in the original HA spectra (Fig. 4e). Similar co-precipitation processes were reported previously^20,35,36,38^.

Only two fluorescent components, i.e. FA-like and protein-like substances, were identified by EEM-PARAFAC analysis in all seven remaining supernatants after each subsequent precipitation step of Fe-FA_P_. In particular, FA in the first four remaining supernatants showed EEM peaks maxima (305–320/440–449 nm for peak M and 250–270/440–449 nm for peak A) that were relatively longer than the three subsequent supernatants (300–305/431–440 and 240–245/431–440 nm, respectively for peak M and peak A) (Fig. 2). These results suggested that the relatively longer excitation-emission wavelength peaks of FA would imply its initially high complexation capacity for Fe^3+^ involving high functionality molecules, whereas the shorter excitation-emission wavelength peaks would be due to molecules featuring a lower functionality. This interpretation is confirmed by earlier studies showing a decrease of the binding capacity of photodegraded FA with respect to original FA^69,70^, and by the well-known blue-shift to shorter excitation-emission wavelengths with respect to the original ones usually caused by photodegradation^71,72^. This interpretation is further supported by the first extracted HA-1 and HA-2 that show excitation-emission peaks longer than the subsequent seven remaining supernatants, which would suggest that, the isolation of Fe-HA_P_ and Fe-FA_P_ from the various environmental samples, no artifact effects would occur on the EEM measurements by the DOM remained in the samples. Differently, peak A and peak T_UV_ of protein-like substances showed shorter wavelength maxima that varied slightly from the first to the seventh remaining supernatant, which might be due to their pH variation between 8.41 and 8.56, as it has been shown for protein-like peaks of extracellular polymeric substances^73^. In conclusion, the preferential formation of stable Fe‒HA_P_ and then Fe‒FA_P_ would confirm the stable sequestration of organic carbon in organic-mineral complexes in the soil matrix^10,13,14,36,74,75^.

### Fe-Tryptophan_P_ and Fe-Tyrosine_P_

Approximately 25.0% and 30.4% of total DOC was precipitated by FeCl_3_ as Fe-Tryptophan_p_ and Fe-Tyrosine_p_ from their corresponding water solutions. Both before and after repeated precipitations the EEM spectra of Fe-Tryptophan_p_ and Fe-Tyrosine_p_ featured the two expected protein-like substances peaks T and T_UV_ (Table S3). Tryptophan and tyrosine are composed of known molecular structures rich in amino carboxylic and hydroxyl functional groups that are able to complex Fe^3+^ ions. These results suggest that low molecular weight organic compounds such as tryptophan and tyrosine could form strong complexes with Fe^3+^ via COOH and OH functional groups of amino acids^76,77^, as it has been shown to occur in surface waters for proteins of extracellular polymeric substances and siderophore complexes of prokaryotes^23–26,42^. The occurrence of such complexation is also evidenced by the FTIR spectra discussed in section "FTIR spectra".

### Elemental composition

The elemental composition of Fe‒DOM_P_ fractions (Table 1) shows that the C% in all Fe‒FA_P_ samples is quite low. In particular, the % of Fe-FA_P_ from river lower than that from lake and soil W_e_, which are all lower than those from soil A_e_ and original and irradiated cow dung samples that are the highest of all Fe-FA_P_ samples. The low C%, especially in some samples, might arise from the low molecular weight (LMW) FA of aqueous samples. The relatively longer Ex/Em of peak M suggests that high molecular weight FA would precipitate initially as Fe-FA_P_ (Fig. 4), whereas the relatively LMW FA would be involved in the last precipitation steps. Obviously, the O% in all Fe‒FA_P_ samples is relatively high and, as a consequence, the C/O ratios are generally quite low, with the lowest values for Fe-FA_P_ from river, lake and soil W_e_, and values ranging from 0.6 to 1.5 for the remaining samples.

**Table 1:** Elemental composition (C, O, N, H, S) (moisture and ash free) and atomic ratios (C/N, C/S, C/H and C/O) of Fe‒fulvic acid precipitates (Fe‒FA_P_) and Fe‒humic acid precipitates (Fe‒HA_P_) from river, lake, soil, original and irradiated cow dung samples, Fe‒tryptophan precipitates (Fe‒TR_P_) and Fe‒tyrosine precipitates (Fe‒TY_P_).

Sample	Source	Ash [%]	C [%]	O [%]	H [%]	N [%]	S [%]	C/N	C/S	C/H	C/O
Fe-FA_P_	Baigou River	1.2	15.5	78.7	1.4	0.4	0.1	47	717	0.9	0.3
Fe-FA_P_	Jingye Lake	1.4	27.7	64.4	4.5	< 0.1	0.1	-	545	0.5	0.6
Fe-FA_P_	W_e_ from forest soil	1.4	27.2	64.3	4.5	< 0.1	< 0.1	-	-	0.5	0.6
Fe-FA_P_	A_e_ from forest soil	1.6	38.0	53.7	2.5	1.5	0.1	29	814	1.3	0.9
Fe-FA_P_	Cow dung original	3.3	46.4	45.1	1.0	2.6	0.1	21	928	3.7	1.4
Fe-FA_P_	Cow dung irradiated	3.5	43.5	47.8	1.7	0.6	1.0	81	118	2.1	1.2
Fe-HA_P_	Forest soil	-	45.1	52.2	1.9	0.8	< 0.1	70	-	2.0	1.2
HA original	"	-	48.5	47.7	1.0	2.1	0.7	27	191	4.1	1.4
Fe-TR_P_	Tryptophan	-	30.6	63.6	3.2	1.0	0.4	35	204	0.8	0.6
Tryptophan original	"	1.2	48.5	47.0	0.5	4.0	< 0.1	14	-	8.3	1.4
Fe-TY_P_	Tyrosine	-	33.8	59.4	2.8	0.9	0.5	44	176	1.0	0.8
Tyrosine original	"	2.6	51.1	45.7	0.6	2.6	< 0.1	23	-	6.9	1.5

Also the H% in Fe-FA_P_ from the river is relatively lower than those in the Fe-FA_P_ from lake and soil W_e_, which are, however, higher than those in samples from soil A_e_ and original and irradiated cow dung samples. Such differences may be due to the different molecular composition of FA (Baluha et al., Stenson et al., Stubbins et al.) isolated using FeCl_3_ from various sources in each extraction process. Differently from the C%, the H% in Fe-HA_P_, Fe-Tryptophan_P_ and Fe-Tyrosine_P_ are higher than those in the corresponding HA, Tryptophan and Tyrosine. Also the atomic C/H ratios are very low for Fe-FA_P_ from river, lake and soil W_e_, and lower than that from soil A_e_. The C/H ratios of Fe-FA_P_ from cow dung and soil HA are higher than those of Fe-FA_P_ from irradiated cow dung and Fe-HA_P_, whereas the C/H ratios of Fe-Tryptophan_P_ and Fe-Tyrosine_P_ are much lower than those of the corresponding Tryptophan and Tyrosine.

The N% features the lowest values in Fe-FA_P_ from river, lake and soil W_e_ and the highest ones in the original HA and Fe-FA_P_ from cow dung, whereas in Fe-FA_P_ from irradiated cow dung sample, Fe-HA_P_, Fe-Tryptophan_P_ and Fe-Tyrosine_P_ the N% is lower than that in the corresponding unreacted sample. The high N% in the original cow dung sample may be attributed to the high content of Tyrosine^65^ that declines upon sunlight exposure of cow dung. The C/N ratio is relatively low in Fe-FA_P_ from soil A_e_, and much higher in irradiated cow dung, Fe-HA_P_, Fe-Tryptophan_P_ and Fe-Tyrosine_P_ than in the corresponding not-irradiated cow dung, soil HA, Tryptophan and Tyrosine. Finally, the S% results ≤ 1.0% in most Fe-FA_P_ samples and only in the Fe^3+^ precipitate from irradiated cow dung sample reaches the maximum value of 1.0%. As expected from the corresponding S%, the C/S ratios of Fe-FA_P_ from cow dung, soil A_e_, and river and lake waters are very high, and those of the remaining samples quite low.

Thus, the Fe-FA_P_ fractions isolated in each step feature a different elemental composition, which is supported by the blue-shift of FA peaks in the RS after each precipitation step (Fig. 2), and confirms previous FTICR-MS studies^78–80^. Further studies on the elemental composition of Fe-FA_P_ isolated in each step will be necessary to achieve a better understanding of FA components and their biogeochemical transformation in specific ecosystems.

### FTIR spectra

The most relevant infrared absorption features and their differences (Fig. 4) are summarized below. (*i*) The presence of two weak bands at 3884–3857 cm^−1^ in Fe-FA_P_ and remaining supernatants from river and lake waters, soil W_e_ and A_e_, and both cow dung samples may be ascribed to aromatic C–H stretching in aromatic ring structures^80^. (*ii*) A band of strong/medium relative intensity at 3400–3450 cm^−1^ appears in the remaining supernatants of Fe precipitates from river and lake waters, soil W_e_ and A_e_, original and irradiated cow dung samples and original soil HA, the relative intensity of which decreases in the corresponding Fe-FA_P_ and Fe-HA_P_ samples. These results would suggest that aromatic OH in FA could effectively donate electrons to *d*-orbitals of Fe^3+^, thereby forming a strong π–d electron bonding system in Fe–O–FA, which, in turn, causes IR absorption to decline or completely disappear. Furthermore, the relatively higher intensity of this band in Fe-FA_P_ from soil A_e_ would suggest that soil FA is richer in electron donating functional groups compared to FA from lake and river waters and cow dung samples^17,65,81–83^, and that many functional groups in soil FA would be unable to donate electrons to the d-orbital of Fe^3+^ possibly due to intermolecular interactions among them^84–86^. (*iii*) The relative intensity of the peaks in the range 1685‒1662 cm^−1^, assigned to aromatic C=C stretching and/or COOH groups, in the remaining supernatants from river and lake waters, soil W_e_ and A_e_ and original and irradiated cow dung samples feature a is slightly lower than that of the corresponding Fe-FA_P_. These results would suggest that these groups could donate electrons to d-orbitals of Fe^3+^ forming a strong π–d electron bonding system Fe–OOC–FA. (*iv*) The peaks at 1523–1500 nm^−1^, assigned to the amide II band, are relatively more intense in Fe-FA_P_ than in the corresponding remaining supernatants from river waters, soil W_e_, and original and irradiated cow dung samples, whereas the opposite occurs for lake and soil A_e_. These results would suggest that the donation of non-bonding electrons from amide II N to d-orbitals of Fe would form either a strong or a weak bonding system in Fe ← :N‒FA_P_. (*v*) A weak peak around 1400 cm^−1^, assigned to C=N stretching of amide I, appears in Fe-FA_P_ from soil W_e_ and original and irradiated cow dung samples, which would imply, similarly to amide II, the formation of a weak π-d electron bonding system between non-bonding electrons of amide I N and d-orbitals of Fe^82,87,88^. (*vi*) A weak peak around 1150 cm^−1^, assigned to S=O and C–O–S stretching of S-containing functional groups, is present in the remaining supernatants from river and lake waters, soil A_e_ and original and irradiated cow dung samples, but it disappears in the corresponding Fe-FA_P_. These results would suggest that S-containing functional groups can donate electrons to d-orbitals of Fe^3+^, forming a π–d electron bonding system in Fe–S–FA_P_. (*vii*) A weak band at 1100–1000 cm^-1^, attributed to C-O stretching of polysaccharides and polysaccharide-like components, is present in Fe-FA_P_ from soil W_e_ and A_e_, irradiated cow dung samples, original HA and the first Fe-HA_P_, but not in the other samples, which would suggest a partial involvement of also these structures in Fe^3+^ complexation.

In general, all FTIR peaks appearing in the spectra of original soil HA precipitated at pH 2 and the first Fe-HA_P_ sample almost disappear in the subsequent Fe-HA_P_ samples, which would imply that all HA functional groups have formed complexes with Fe^3+^. Also for tryptophan and tyrosine all FTIR peaks are strongly reduced in intensity or disappear in Fe-Tryptophan_P_ and Fe-Tyrosine_P_, which confirms that the functional groups of both compounds are capable of forming strong complexes with Fe^3+^.

In conclusion, FTIR results discussed above suggest that either strong or weak π‒d electron bonding systems can be formed in Fe-FA_P_, Fe-HA_P_, and Fe-Tryptophan_P_ and Fe-Tyrosine_P_ by electron donation from the functional groups of the corresponding original samples.

### Mechanisms for Fe-DOM_P_ formation and isolation

**Figure 5:**
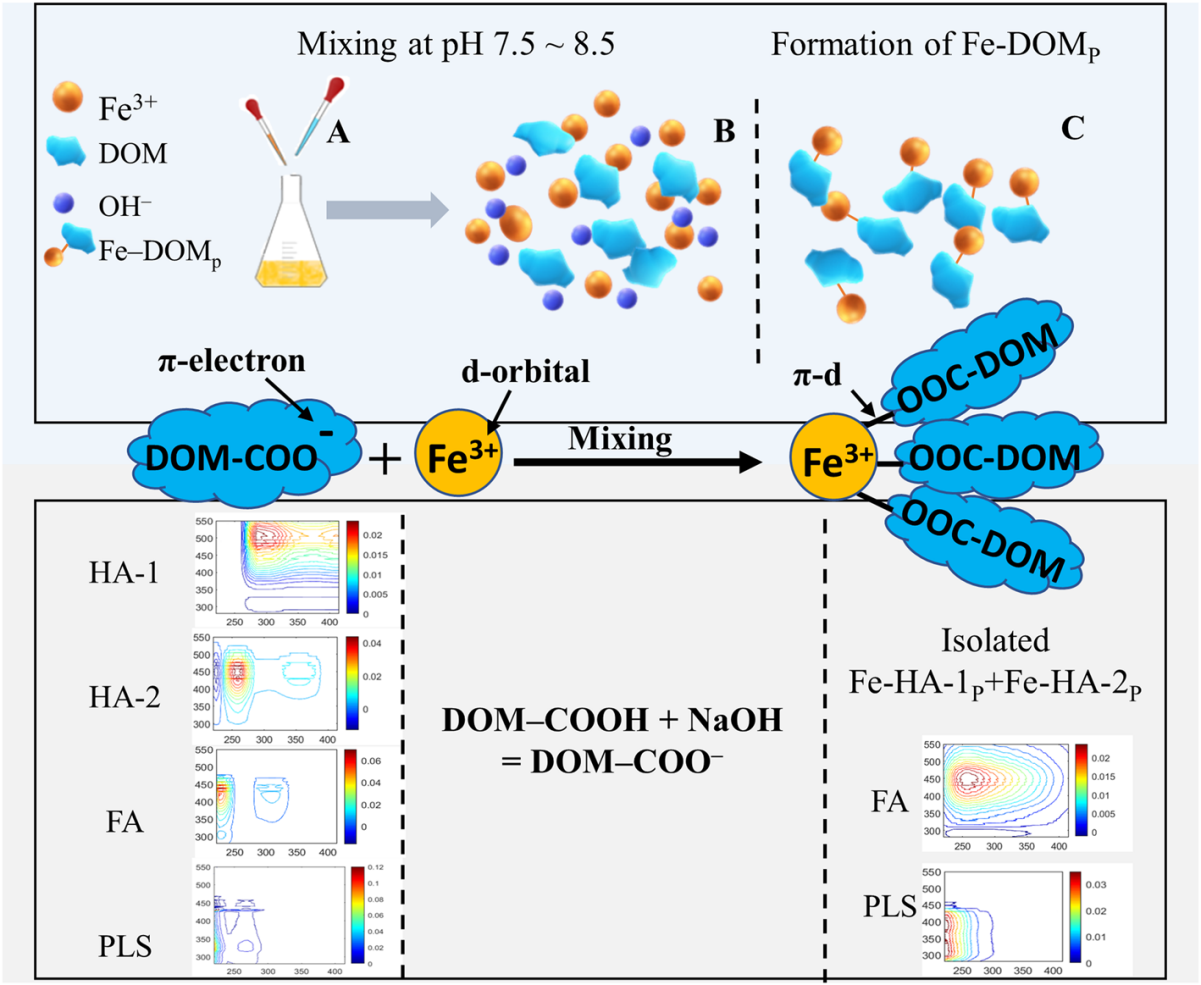
Conceptual model of the formation of Fe‒dissolved organic matter (DOM) precipitates (Fe‒DOM_P_) after subsequent additions of aqueous solutions of FeCl_3_ at pH 7.5‒8.5. (**A**) FeCl_3_ was slowly added to the pH adjusted /7.5 ~ 8.5) DOM solutions with simultaneous addition of NaOH to keep the pH at 7.5–8.5, under gentle magnetic stirring; (**B**) the reaction system was mixed and allowed to react at pH 7.5 ~ 8.5; (**C**) supersaturation initiated the formation of Fe‒DOM_P_, after 5–10 min Fe‒DOM_P_ settled, then the solution was centrifuged, and finally Fe‒DOM_P_ was isolated, freeze-dried and stored with the supernatant remaining solution (RS) for further analysis.

The conceptual model of the processes of formation and isolation of Fe‒DOM_P_ (Fig. 5) illustrates how Fe‒DOM_P_ complexes are constantly formed from DOM components and Fe^3+^ ions in aqueous, slightly alkaline conditions (pH 7.5–8.5). Specifically, DOM functional groups, such as carboxylate (‒COO^−^), hydroxylate (‒O^‒^) and thiol (‒SH) groups, rapidly deprotonate in these conditions, thus determining the formation of Fe‒DOM_P_ involving strong or weak π–d electron bonding systems by donation of electrons from deprotonated electron-rich functional groups of DOM to the unpaired d-orbitals of Fe^3+^^31,89^. Moreover, the π–d electron bonding system in Fe–DOM would be stabilized by the lower energy of the high spin state due to its greater electron-nucleus attraction^90^, which would yield a strong binding of Fe^3+^ to HS and DOM, so that Fe^3+^ results widely distributed in the larger molecular size fractions^91^.

The π–d electron bonding system occurring between Fe^3+^ and DOM^92^ can be outlined in the following two reactions:

$$2{FeCl}_{3}+6NaOH+6DOM-COOH=2{Fe}^{3+}+6DOM-{COO}^{-}+6{NaCl}+6{H}_{2}O$$

$$2{Fe}^{3+}+6DOM-{COO}^{-}={2Fe(OOC-DOM)}_{3}$$

Finally, the different electron donation capacity/efficiency of the various DOM functional groups to unpaired d-orbitals of Fe^3+^ would be the determining factor for the formation of either strong or weak complexation in the various Fe‒DOM_P_ samples. Notably, the anionic forms (‒O^−^ or ‒COO^−^) of HS functionalities (phenolic OH and carboxylic acid-COOH, respectively) would be favored in donating π-electrons to Fe^3+^d-orbitals due the high availability of Fe^3+^ before reaching the saturation state during its addition in the precipitation process. The achieved Fe‒DOM_P_ might be comparable with both Fe-(oxy)hydroxides (FeOOH) in either dissolved or colloidal state in water^31,52,93–95^, and Fe-(oxy)hydroxide minerals and organo-mineral complexes in soils and sediments^10,12,13,96–101^. In particular, Fe-DOM_P_ would include, besides organo-Fe(OH)_3_ complexes, also organo-Fe(III) oxyhydroxides and organo-Fe(III) minerals^102^. Moreover, Fe-DOM_P_ might undergo microbial reduction either biologically or by conversion to organo-Fe(II) oxyhydroxides under diverse environmental conditions^103–107^. Finally, Fe(II) oxyhydroxide (FeOOH) forms large aggregates in alkaline conditions at pH > 7 using Fe(II)-salts in the absence of DOM/HS^52^, which is very similar to the formation of Fe-DOM_P_ using Fe(III)-salts in the presence of DOM/HS in this work. Therefore, Fe-DOM_P_/Fe(III) oxyhydroxides and Fe(II) oxyhydroxides would be formed depending on the occurrence of Fe(III) or Fe(II) in the diverse environments, including soils, sediments and surface waters^107–113^.

### Environmental and practical implications

The precipitation of Fe-DOM_P_ in slightly alkaline conditions would suggest that DOM fractions can exist in a strong binding state with Fe^3+^ in slightly alkaline surface waters, soil and other environmental systems such as rainwater, groundwater, glaciers, aerosols, etc. Such conditions are crucial for the fate of DOM and Fe-DOM_P_, i.e., their solubility, speciation, reactivity, degradation, redox reactions, bioavailability and transport^19,21,29,31,114^. Differently, DOM in surface waters having a neutral to acidic pH would show less probability to be complexed by metal ions such as Fe^3+^.

Noteworthy, the observation that approximately 3.0 mL of FeCl_3_ (30 g L^−1^) solution is needed to ~ 900 mL of a water sample to start the precipitation of Fe-FA_P_ implies that up to ~ 2.99 mL Fe-DOM complexes remain in a soluble state in surface waters, and that dissolved iron is removed only at concentrations greater than its apparent solubility^115^. This situation is very similar to that of Fe-(oxy)hydroxides in freshwaters and seawaters either in the dissolved or colloidal state^31,52,93–95,116,117^. Moreover, Fe^3+^ in oxygenated seawater at pH ~ 8 is thermodynamically stable in the form of Fe(III) hydroxide complexes, which have a high possibility to be scavenged by sinking particulate matter and are in equilibrium with Fe(III) oxyhydroxide particulates characterized by low solubility^118^. Furthermore, Fe^3+^-DOM complexes are known to be the primary responsible of the occurrence of photo-Fenton reactions in DOM degradation under sunlit surface waters^31,95,114^, where Fe oxyhydroxide are involved in reactions involving O_2_^**·**−^ and ligand-to-metal charge transfer^95^.

Furthermore, various siderophores enable the acquisition of Fe from its minerals (e.g. oxides, hydroxides, goethite) and organic complexes under various environmental conditions^119–123^. Siderophores are relatively low molecular weight organic substances that can bind up to 99% of Fe dissolved in seawater^124^ and may be compared with Fe-Tryptophan_P_ and Fe-Tyrosine_P_. In particular, siderophore-bound Fe is utilized by marine bacteria and eukaryotic phytoplankton as a sink in seawaters^23–26,42,125–128^. Furthermore, the primary factor that affects the up to 99% of Fe^3+^ to remain in a complex state with DOM in seawaters^32,33^ can be ascribed to their common slightly alkaline conditions (average pH = 8.07 ± 0.02)^66^. Finally, these phenomena would lead to a long-term preservation of marine DOM that can be partly transferred from surface to deep waters where it can be sequestered over thousand-year timescales^129^.

On the other hand, the Fe^3+^ capacity to form insoluble complexes with soil DOM fractions provides evidence of sequestration and sedimentation of DOM, and its subsequent long-term stabilization as previously reported^10–14,74,75^. The results of this work show that Fe-HA_P_ are rapidly precipitated from the forest soil HA due to its macromolecular size, and then Fe-FA_P_ are formed from the remaining soil HA solution due to smaller size of FA (Fig. 2). This would imply that Fe-HA_P_/Fe-FA_P_, similarly to Fe-(oxy)hydroxide minerals and organo-mineral complexes, can stabilize SOM in soils and sediments^10,12,13,74,96–101,112^. The existence of strong complexes of HA/FA in Fe-DOM_P_ is further supported by a recent study showing that Fe (oxy)hydroxides generally exert a stronger impact on SOC storage and stabilization than aluminous clays^112^. These results also suggest that Fe-(oxy)hydroxide minerals and organo-mineral complexes consist of strong complexes formed by donation of electrons from functional groups to d-orbitals of Fe^3+^ which result in strong π-d electron bonding systems the existence of which was not completely clarified earlier^10,12,13,99,130,131^. The new results of this work are thus expected to provide new insights and a better understanding of soil Fe minerals and organo-mineral complexes, as well as their behaviors not only in soils/sediments, but also in surface waters where they are introduced from soils^115,116,132^. In particular, the Fe precipitation technique might be applied efficiently to isolate specific DOM/HS components from specific Fe minerals and clay mineral after their dissolution in alkaline or acidic media and then assess its biogeochemical state in the minerals.

In conclusion, the Fe precipitation method allows a very high recovery of HA and FA, approximately 76.2% by a single precipitation from soil HA and 38.6–93.8% from the environmental samples. Therefore, the Fe precipitation method would represent an effective/useful procedure for extracting/isolating humic substances rather than the entire DOM from aqueous media in the laboratory or for industrial purposes. The novel approach proposed and developed in this work appears robust and promising in opening a new frontier for the isolation of even tiny DOM amounts from any environmental sample, also considering that FeCl_3_ is easily available, very cheap and free of organic contaminants and can replace the currently used organic-based resins.

## Supplementary Information

Supplementary Information.

## Data Availability

All data and materials have been provided in this manuscript and also supplementary material.
