# Author Correction: Isolation of dissolved organic matter from aqueous solution by precipitation with FeCl_3_: mechanisms and significance in environmental perspectives

**DOI:** 10.1038/s41598-023-32252-w

**Published:** 2023-03-28

**Authors:** Jie Zhang, Khan M. G. Mostofa, Xuemei Yang, Mohammad Mohinuzzaman, Cong‑Qiang Liu, Nicola Senesi, Giorgio S. Senesi, Donald L. Sparks, H. Henry Teng, Longlong Li, Jie Yuan, Si‑Liang Li

**Affiliations:** 1 Institute of Surface‑Earth System Science, School of Earth System Science, Tianjin University, 92 Weijin Road, Tianjin, 300072 China; 2 Tianjin Key Laboratory of Earth Critical Zone Science and Sustainable Development in Bohai Rim, Tianjin University, 92 Weijin Road, Tianjin, 300072 China; 3 Institute of Ecology, College of Urban and Environmental Sciences, Peking University, Beijing, China; 4 Department of Environmental Science and Disaster Management, Noakhali Science and Technology University, Noakhali, Bangladesh; 5 Dip.to di Scienze del Suolo, Della Pianta e degli Alimenti, Università Degli Studi Di Bari “Aldo Moro”, Via G. Amendola 165/A, 70126 Bari, Italy; 6 CNR - Istituto per la Scienza e Tecnologia dei Plasmi (ISTP) - Sede Di Bari Via Amendola, 122/D, 70126 Bari, Italy; 7 Department of Plant and Soil Sciences, Delaware Environmental Institute, University of Delaware, Newark, DE 19716‑7310 USA; 8 College of Resources and Environment, Xingtai University, Quanbei East Road 88, Qiaodong District, Xingtai City, Hebei Province China; 9 Haihe Laboratory of Sustainable Chemical Transformations, Tianjin, 300192 China

Correction to: *Scientific Reports* 10.1038/s41598-023-31831-1, published online 20 March 2023

The original version of this Article contained errors in the Results and discussion, under the subheading ‘Mechanisms for Fe-DOMP formation and isolation’,

“2*FeCl*_3_ + 6*NaOH* + 6*DOM* − *COOH* = 2*Fe*^3^ + 6*DOM* − *COO*^−^ + 2*NaCl*_3_ + 6*H*_2_*O*.”

now reads:

“2*FeCl*_3_ + 6*NaOH* + 6*DOM* − *COOH* = 2*Fe*^3^ + 6*DOM* − *COO*^−^ + 6*NaCl* + 6*H*_2_*O*.”

The original Article has been corrected.

