# Supplementary material for: Isolation of dissolved organic matter from aqueous solution by precipitation with FeCl3: mechanisms and significance in environmental perspectives
Source: Sci Rep. 2023 Mar 20;13:4531. doi: 10.1038/s41598-023-31831-1 (PMC10027667; doi:10.1038/s41598-023-31831-1)
Supplement: Supplementary file 1 — Supplementary Information. [file 41598_2023_31831_MOESM1_ESM.docx]

Isolation of dissolved organic matter from aqueous solution by precipitation with FeCl_3_: Mechanisms and significance in environmental perspectives

*Jie Zhang^1,2^, Khan M.G. Mostofa^1,2 *^, Xuemei Yang^1,3^, Mohammad Mohinuzzaman^1,4^, Cong-Qiang Liu^1,2^, Nicola Senesi^5^, Giorgio S. Senesi^6^, Donald L. Sparks^7^, H. Henry Teng^1,2^, Longlong Li^1^, Jie Yuan^8^, and Si-Liang Li^1,2,9 *^*

^1^ Institute of Surface-Earth System Science, Tianjin University, 92 Weijin Road, Tianjin 300072, China.

^2^ Tianjin Key Laboratory of Earth Critical Zone Science and Sustainable Development in Bohai Rim, Tianjin University, 92 Weijin Road, Tianjin 300072, China

^3^ Institute of Ecology, College of Urban and Environmental Sciences, Peking University, Beijing, China

^4^ Department of Environmental Science and Disaster Management, Noakhali Science and Technology University, Noakhali, Bangladesh

^5^ Dip.to di Scienze del Suolo, della Pianta e degli Alimenti, Università degli Studi di Bari "Aldo Moro", Via G. Amendola 165/A, 70126 BARI – Italy.

^6^ CNR - Istituto per la Scienza e Tecnologia dei Plasmi (ISTP) - sede di Bari Via Amendola, 122/D - 70126 Bari, Italy.

^7^ Delaware Environmental Institute, Department of Plant and Soil Sciences, University of Delaware, Newark, Delaware 19716-7310, United States

^8^ College of Resources and Environment, Xingtai University, Quanbei East Road 88, Qiaodong District, Xingtai City, Hebei Province.

^9^ Haihe Laboratory of Sustainable Chemical Transformations, Tianjin 300192, China.

*Email: [mostofa@tju.edu.cn](mailto:mostofa@tju.edu.cn) (Khan M.G. Mostofa), [siliang.li@tju.edu.cn](mailto:siliang.li@tju.edu.cn) (Si-Liang Li)

Number of Pages: 12

Number of Tables: 3

Number of Figures: 4

**Table S1.** Dissolved organic carbon (DOC) concentrations (in ppm) in original samples after filtration and percentages (%) in the various precipitates with the estimated contribution of HA.

| Sample | DOC | FeCl_3_ precipitates | Contribution of HA |
| --- | --- | --- | --- |
|  | (ppm) | (%) | (%) |
| **Baigou River** |  |  |  |
| Initial DOC | 3.9±0.04 |  |  |
| DOC after precipitation of HA at pH 2 | 3.6±0.02 |  | 6.4 |
| DOC after first precipitation of Fe-FA_P_ | 2.0±0.11 | 44.6 |  |
| DOC after second precipitation of Fe-FA_P_ | 1.0±0.16 | 42.8 |  |
| **Jingye Lake** |  |  |  |
| Initial DOC | 5.3±0.04 |  |  |
| DOC after precipitation of HA at pH 2 | 3.7±0.07 |  | 29.5 |
| DOC after precipitation of Fe-FA_P_ | 2.3±0.02 | 38.6 |  |
| **Forest soil** We |  |  |  |
| Initial DOC | 7.9±0.04 |  |  |
| DOC after precipitation of HA at pH 2 | 4.8±0.06 |  | 40.0 |
| DOC after precipitation of Fe-FA_P_ | 1.3±0.18 | 72.9 |  |
| **Forest soil Ae** |  |  |  |
| Initial DOC | 35.2±0.09 |  |  |
| DOC_CS_ after precipitation of HA at pH 2 | 27.9±0.04 |  | 20.8 |
| DOC_CS_ after precipitation of Fe-FA_P_ | 5.0±0.05 | 82.1 |  |
| **Cow dung extract original** |  |  |  |
| Initial DOC | 1306.6±0.04 |  |  |
| DOC_CS_ after precipitation at pH 2 | 1382.2±0.04 |  |  |
| DOC_CS_ after precipitation of Fe-FA_P_ | 107.2±0.09 | 92.2 | NA* |
| **Cow dung extract after irradiation** |  |  |  |
| Initial DOC | 1018.3±0.01 |  |  |
| DOC after precipitation at pH 2 | 1181.1±0.05 |  |  |
| DOC after precipitation of Fe-FA_P_ | 72.9±0.03 | 93.8 | NA |
| **Forest soil HA** |  |  |  |
| Initial DOC | 11.4±0.02 |  |  |
| DOC after first precipitation of Fe‒HA_P_ | 2.7±0.02 | 76.2 |  |
| DOC after seventh precipitation of Fe‒HA_P_ | 1.6±0.04 | 40.9 |  |
| **Tryptophan (TR)** |  |  |  |
| DOC before adding FeCl_3_ | 57.0±0.02 |  |  |
| DOC after final precipitation of Fe-TR_P_ | 42.7±0.01 | 25.0 |  |
| **Standard tyrosine (TY)** |  |  |  |
| DOC before adding FeCl_3_ | 58.7±0.01 |  |  |
| DOC after final precipitation of Fe-TR_P_ | 40.8±0.02 | 30.4 |  |

*NA means not applicable due to absence of HA in cow dung.

**Table S2.** Wavelengths and intensities of fluorescence peak M of fulvic acids (FA) in the remaining solution (RS) after subsequent precipitations of Fe-FA_P_.

| Sample | Precipitation | Peak M | Fluorescence intensity |
| --- | --- | --- | --- |
|  | steps | Ex/Em (nm) | (arbitrary units) |
| **Forest soil** |  |  |  |
| W_e_ adjusted at pH ~7.5 | | 310/416 | 800.8 |
| " | 1st | 308/409 | 437.1 |
| " | 2nd | 305/403 | 370 |
| " | 3rd | 300/400 | 323.3 |
| " | 4th | 300/401 | 243.5 |
| " | 5th | 300/397 | 231.6 |
| " | 6th | 295/397 | 219.5 |
| " | 7th | 295/394 | 217.6 |
| " | 8th | 300/399 | 195.1 |
| " | 9th | 310/396 | 182.5 |
| " | 10th | 305/397 | 184.8 |
| " | 11th | 300/395 | 178.4 |
| A_e_ adjusted pH ~7.5 | | 310/412 | 2076 |
| " | 1st | 310/410 | 1699 |
| " | 2nd | 305/406 | 1874 |
| " | 3rd | 300/403 | 1619 |
| " | 4rth | 300/401 | 1629 |
| " | 5th | 305/398 | 1586 |
| " | 6th | 300/398 | 1472 |
| " | 7th | 304/392 | 1479 |
| " | 8th | 305/397 | 1187 |
| " | 9th | 310/393 | 1367 |
| " | 10th | 306/393 | 1202 |
| " | 11th | 305/396 | 1031 |

Table S2 (Continued)

| **Baigou river water** | |  |  |
| --- | --- | --- | --- |
| Adjusted at pH ~7.5 | | 315/409 | 132.8 |
| " | 1st | 315/409 | 113.5 |
| " | 2nd | 315/408 | 109.1 |
| " | 3rd | 315/403 | 108.4 |
| " | 4th | 315/408 | 103.3 |
| " | 5th | 315/408 | 103 |
| " | 6th | 315/410 | 100.9 |
| " | 7th | 315/409 | 98.28 |
| " | 8th | 315/408 | 95.23 |
| " | 9th | 315/408 | 97.78 |
| " | 10th | 315/408 | 95.25 |
| " | 11th | 315/405 | 94.15 |
| " | 12th | 315/406 | 91.91 |
| " | 13th | 315/407 | 89.67 |
| " | 14th | 315/407 | 90.61 |
| " | 15th | 315/406 | 90.79 |
| " | 16th | 315/407 | 87.24 |
| **Jingye Lake water** | |  |  |
| Adjusted at pH ~7.5^*^ | |  |  |
| " | 1st | 290/375 | 355.4 |
| " | 2nd | 290/369 | 334.4 |
| " | 3rd | 290/377 | 313.2 |
| " | 4th | 285/375 | 305.5 |
| " | 5th | 290/383 | 293 |
| " | 6th | 290/383 | 274.2 |
| " | 7th | 290/386 | 276.4 |

Table S2 (Continued)

| **Cow dung original** | |  |  |
| --- | --- | --- | --- |
| Diluted 50 times and adjusted at pH ~8.5^*^ | | 315/411 | 907.3 |
| " | 1st | 315/412 | 818.1 |
| " | 2nd | 315/415 | 757.2 |
| " | 3rd | 315/414 | 696.2 |
| " | 4th | 315/412 | 633.1 |
| " | 5th | 315/412 | 536.7 |
| " | 6th | 315/411 | 549.2 |
| " | 7th | 315/407 | 503.9 |
| " | 8th | 315/412 | 433.2 |
| " | 9th | 315/405 | 473.5 |
| " | 10th | 315/407 | 487.6 |
| " | 11th | 315/411 | 450 |
| " | 12th | 315/408 | 435 |
| " | 13th | 315/406 | 406.9 |
| " | 14th | 315/407 | 393.5 |
| " | 15th | 315/402 | 393.3 |
| " | 16th | 315/403 | 355.5 |
| **Cow dung irradiated** | |  |  |
| Diluted 30 times and adjusted at pH ~8.5^*^ | | 315/409 | 1374 |
| " | 1st | 315/410 | 1183 |
| " | 2nd | 315/411 | 1112 |
| " | 3rd | 315/414 | 881.6 |
| " | 4th | 315/408 | 875.1 |
| " | 5th | 315/410 | 778.7 |
| " | 6th | 315/415 | 708.8 |
| " | 7th | 315/413 | 666.3 |
| " | 8th | 315/410 | 661.9 |

Table S2 (Continued)

| " | 9th | 315/408 | 715 |
| --- | --- | --- | --- |
| " | 10th | 315/404 | 683.3 |
| " | 11th | 315/408 | 676.1 |
| " | 12th | 315/406 | 653.3 |
| " | 13th | 315/405 | 605.9 |
| " | 14th | 315/405 | 582.7 |
| " | 15th | 315/402 | 576.7 |
| " | 16th | 312/402 | 514.9 |

**Table S3.** Fluorescent components and their EEM peak maxima identified by the PARAFAC model in the fluorescence EEM spectra of the various samples before and after precipitation with FeCl_3_ and the corresponding remaining solutions (RS).

Table S3 (continued)

**Figure S1.** Flow diagram of the extraction procedure of soil dissolved organic matter (DOM) fractions. After grinding the soil samples, Milli-Q water and NaOH aqueous solution were successively used to extract DOM, and two soil DOM extracts were obtained, which were soil water extract (W_e_) and soil alkaline extract (A_e_), respectively.


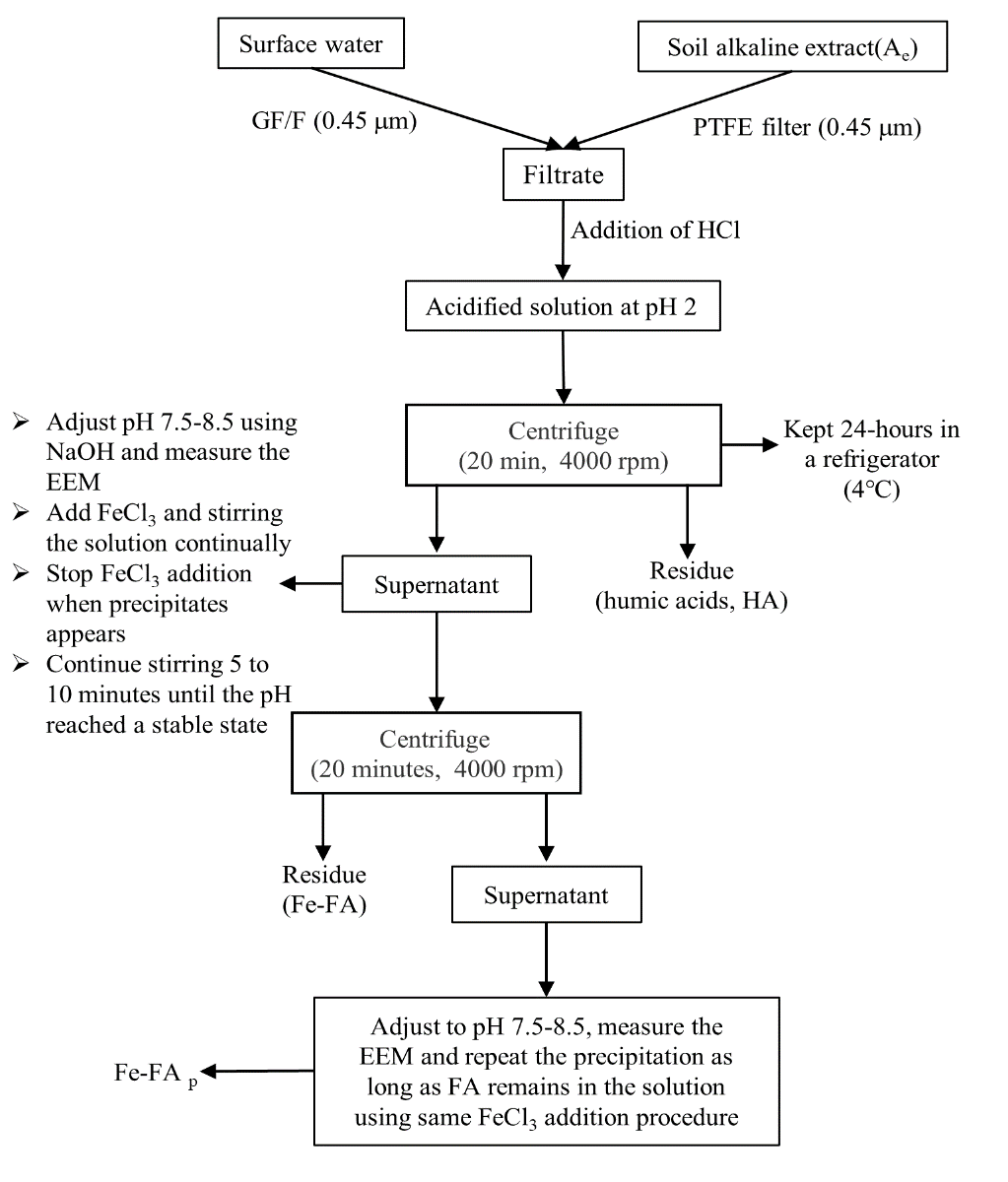


**Figure S2.** Flow diagram of the isolation/precipitation procedure followed to obtain the Fe-FA precipitates (Fe-FA_P_) fractions. First, the HA in the sample was removed by adjusting the pH value of the sample to about 2; After that, Fe-FA_P_ fractions were obtained by adjusting the pH value of the sample to about 7.5~8.5 and adding FeCl_3_ solution at the same time.


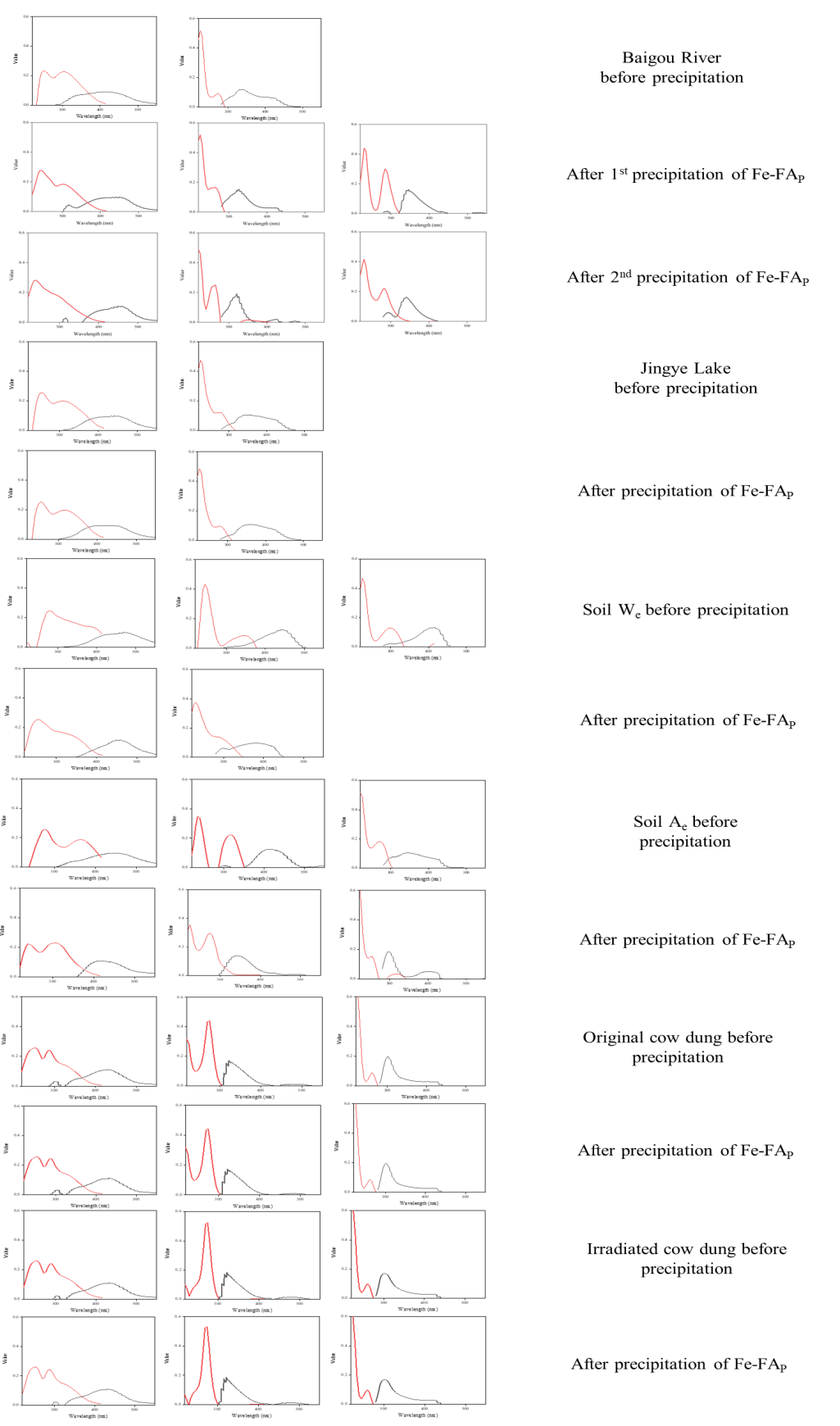


**Figure S3.** Loading figures of the fluorescent components identified by the EEM-PARAFAC model in all individual aqueous samples and their remaining solutions (RS) after each repeated precipitation of Fe-FA_P_.


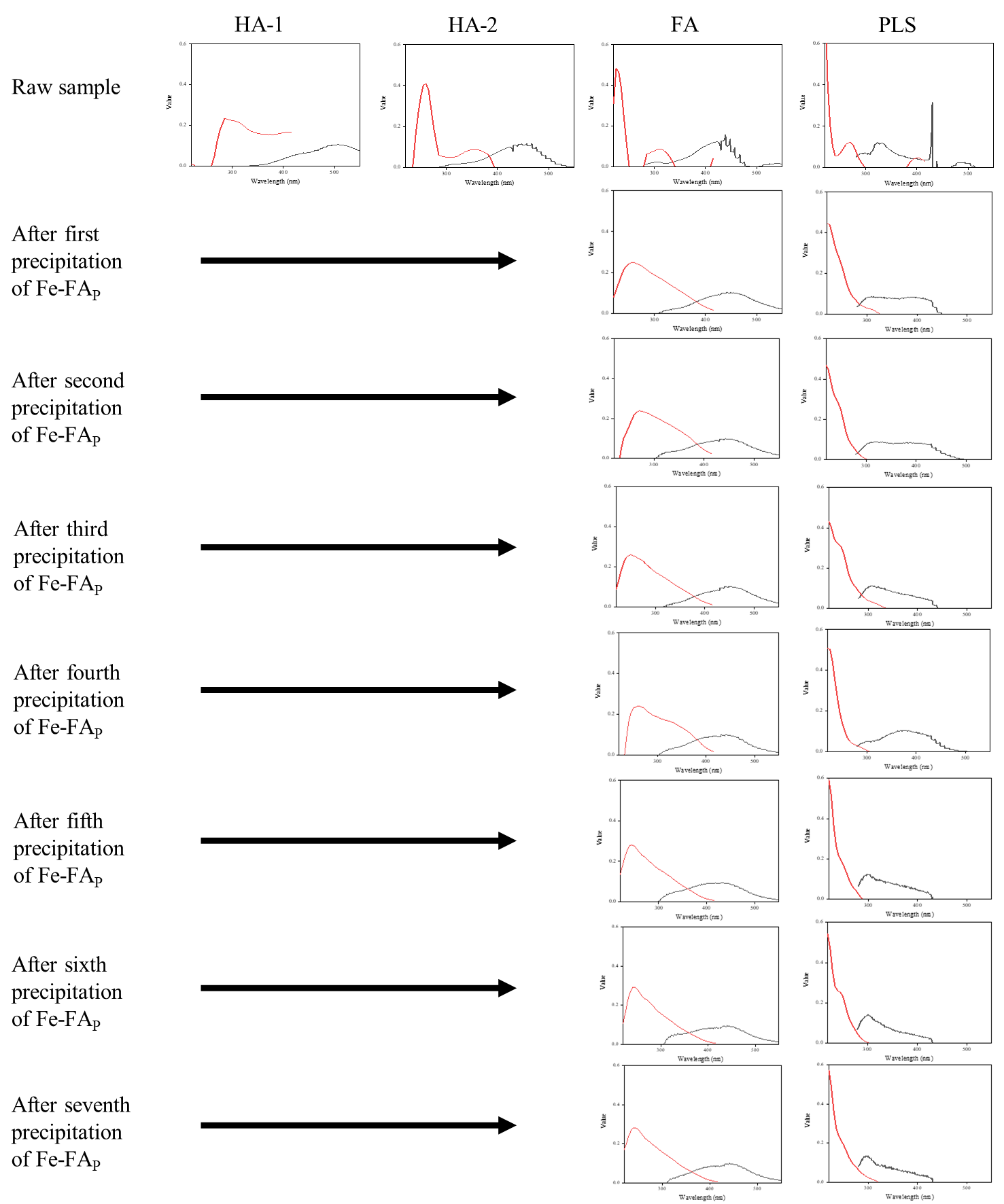


**Figure S4.** Loading figures of the fluorescent components identified by the EEM-PARAFAC model in humic acids (HA) and their remaining solutions (RS) after each repeated precipitation of Fe-HA_P._
